# Comparison of intraocular lens power calculation formulas in patients with a history of acute primary angle-closure attack

**DOI:** 10.1186/s12886-023-03232-5

**Published:** 2023-11-24

**Authors:** Na Hyun Kim, Yujin Gim, Kyu-Ryong Choi, Wool Suh, Roo Min Jun, Kyung Eun Han

**Affiliations:** 1 The Institute of Ophthalmology and Optometry, Department of Ophthalmology, Ewha Womans University College of Medicine, Mokdong Hospital, Seoul, South Korea; 2 The Institute of Ophthalmology and Optometry, Department of Ophthalmology, Ewha Womans University College of Medicine, Seoul Hospital, Seoul, South Korea

**Keywords:** Acute primary angle closure, Axial length, Intraocular lens calculation formula, Laser peripheral iridotomy, Prediction

## Abstract

**Background:**

To compare the accuracy of nine intraocular lens (IOL) power calculation formulas, including three traditional formulas (SRK/T, Haigis, and Hoffer Q) and six new-generation formulas (Barrett Universal II [BUII], Hill-Radial Basis Function [RBF] 3.0, Kane, Emmetropia verifying optical [EVO], Ladas Super, and Pearl-DGS) in patients who underwent cataract surgery after acute primary angle closure (APAC).

**Methods:**

In this retrospective cross-sectional study, 44 eyes of 44 patients (APAC) and 60 eyes of 60 patients (control) were included. We compared the mean absolute error, median absolute error (MedAE), and prediction error after surgery. Subgroup analyses were performed on whether axial length (AL) or preoperative laser peripheral iridotomy affected the postoperative refractive outcomes.

**Results:**

In the APAC group, all formulas showed higher MedAE and more myopic shift than the control group (all P < 0.05). In APAC eyes with AL ≥ 22 mm, there were no differences in MedAEs according to the IOL formulas; however, in APAC eyes with AL < 22 mm, Haigis (0.49 D) showed lower MedAE than SRK/T (0.82 D) (P = 0.036) and Hill-RBF 3.0 (0.54 D) showed lower MedAE than SRK/T (0.82 D), Hoffer Q (0.75 D) or Kane (0.83 D) (P = 0.045, 0.036 and 0.027, respectively). Pearl-DGS (0.63 D) showed lower MedAE than Hoffer Q (0.75 D) and Kane (0.83 D) (P = 0.045 and 0.036, respectively). Haigis and Hill-RBF 3.0 showed the highest percentage (46.7%) of eyes with PE within ± 0.5 D in APAC eyes with AL < 22 mm. Iridectomized eyes did not show superior precision than the non-iridotomized eyes in the APAC group.

**Conclusions:**

Refractive errors in the APAC group were more myopic than those in the control group. Haigis and Hill-RBF 3.0 showed high precision in the eyes with AL < 22 mm in the APAC group.

**Supplementary Information:**

The online version contains supplementary material available at 10.1186/s12886-023-03232-5.

## Background

Acute primary angle closure (APAC) is an ophthalmological emergency that causes extreme intraocular pressure (IOP) rise with blurred vision accompanied by severe eye pain. Most patients with a history of APAC have vulnerable anatomical features, such as anteriorly positioned and thickened lenses, short axial length (AL), shallow anterior chamber depth (ACD), or narrow angular structures. In addition to the pupillary block factor, non-pupillary block factors such as plateau iris syndrome, lens-related factors, or retrolental factors may affect the APAC crisis. In most of the eyes, more than one mechanism may be involved in the pathogenesis of angle closure [1].

For the treatment of APAC, cataract extraction is an effective choice for controlling IOP and restoring the anterior chamber structure by removing both lens-related and pupillary block factors [2–4]. However, the risk of complications for cataract surgery in these patients is relatively high due to the shallow anterior chamber and lens instability caused by the zonular weakness [5]. Furthermore, most studies reported that refractive outcomes may be inaccurate in APAC patients [1, 6–8]. Regarding conventional IOL formulas, different results were reported according to the studies: more myopic shift for the SRK/T formula and hyperopic shift for the Hoffer Q formula [8, 9].

Next-generation IOL formulas such as Barrett Universal II (BUII) or Kane formula have been shown to have better or comparable predictability than conventional IOL formulas in routine cataract surgery [10]. Newly developed IOL formulas, including former ones, use various principles and variables such as ACD or lens thickness (LT) in addition to the keratometry (K) and AL; however, these variables may be distorted and abnormally related to other anatomic structures in APAC patients. In these specific circumstances, refractive outcomes using newer IOL formulas must be validated. Recently, Hou et al. reported that among 7 IOL formulas, including new-generation formulas such as BUII, Kane, and Emmetropia verifying optical [EVO] formulas, Kane formula showed superior refractive outcomes than others [7]. However, in their study, patients diagnosed with APAC and primary angle closure glaucoma (PACG) were included, various types of IOL were inserted, and combined surgery, such as trabeculectomy or surgical peripheral iridectomy, was performed in some patients. Another recent study compared accuracy of 6 formulas (BUII, Haigis, Hill-Radial Basis Function [RBF] 3.0, Hoffer Q, Kane, and Sanders Retzlaff Kraff/Theoretical [SRK/T] in APAC using a single type of IOL and concluded that Kane, Hill-RBF and SRK/T showed higher accuracy [11]. In our study, we compared the prediction errors between nine IOL power calculation formulas (SRK/T, Haigis, Hoffer Q, BUII, Hill-RBF 3.0, Kane, EVO, Ladas Super, and Pearl-DGS formulas) in patients who underwent cataract surgery using a single type of IOL who experienced APAC with additional studies on the effect of axial length of the eye and the performance of a laser peripheral iridotomy (LPI).

## Materials and methods

### Subjects

This retrospective study was approved by the Institutional Review Board (No. EUMC 2022-01-015) of Ewha Womans University Mokdong Hospital. The waiver of consent was obtained due to the minimal risk with personal information protection measures in place. This research has followed the tenets of the Declaration of Helsinki.

Medical records were reviewed for this retrospective cross-sectional study between January 2012 and July 2021 at Ewha Womans University Mokdong Hospital. Patients who underwent unilateral phacoemulsification with IOL implantation into the capsular bag were included. APAC was defined when typical APAC-related symptoms such as ocular pain, blurred vision, nausea, vomiting or headache and appositional or synechial closure, leading to IOP elevation > 30 mmHg, occurred. Subgroup analyses were performed to evaluate whether the AL or preoperative LPI affects postoperative refractive outcomes in the APAC group. The control group included patients with no history of other ocular diseases except cataracts. Patients who received intracapsular cataract extraction, extracapsular cataract extraction, or IOL implantation in the sulcus or scleral fixation were excluded from both groups. In the APAC group, patients who had other ocular diseases such as neovascular glaucoma, uveitic glaucoma, pseudoexfoliation syndrome, and any other retinal or corneal diseases and patients with a history of ocular trauma or other ophthalmic surgeries such as refractive surgery or vitrectomy were also excluded.

### Surgical procedure

Cataract surgery was performed in a standard manner under topical anesthesia in the sequence of 2.2- or 2.7-mm clear corneal incision, continuous curvilinear capsulorrhexis, hydrodissection and or hydrodelineation, phacoemulsification, followed by IOL implantation into the capsular bag. All surgeries were uneventfully performed by one of three experienced surgeons (K. R. C., R. M. J., or K. E. H.). A one-piece monofocal IOL (Tecnis ZCB00, Johnson & Johnson Vision, Santa Ana, CA, USA) were implanted for all eyes.

### Biometry measurement and formulas

Preoperative AL, K, ACD, and white-to-white (WTW) were measured using IOL master 500 (Carl Zeiss Meditech, Jena, Germany). The optimal IOL power was calculated using the following nine formulas: SRK/T, Haigis, Hoffer Q, BUII, Hill-RBF 3.0, Kane, EVO, Ladas super, and Pearl-DGS. Unlike the traditional formulas (SRK/T, Haigis, and Hoffer Q) provided by IOL master 500, the calculation with new generation formulas (BUII, Hill-RBF 3.0, Kane, EVO, Ladas Super, and Pearl-DGS) was not available by the device. Therefore, the biometrics obtained by IOL master 500 were entered on the following internet sites using the latest updated version: http://calc.apacrs.org/barrett_universal2105 for the BUII, https://rbfcalculator.com/ for calculation for the Hill-RBF 3.0, https://www.iolformula.com for Kane, https://www.evoiolcalculator.com/calculator.aspx for EVO formula, and http://iolcalc.com for Ladas super formula and https://iolsolver.com for Pearl-DGS. Optimized lens constants were used as follows: 119.3 (A-constant) for SRK/T, EVO, and Ladas super, 119.39 (A-constant) for BUII, 119.34 (A-constant) for Hill-RBF, 119.36 (A-constant) for Kane, 119.35 for Pearl-DGS, -1.302, 0.210, and 0.251 (a0, a1, and a2) for Haigis, and 5.79 (pACD) for Hoffer Q. The optimized constants were downloaded from the Users Group for Laser Interference Biometry (ULIB, http://ocusoft.de/ulib/c1.htm) or offered by the online calculators.

Refractive prediction error was compared using the absolute error (diopter [D]), which was calculated by the absolute value of the difference between postoperative spherical equivalent (SE) and the predicted refractive power calculated by each formula (AE, i.e., the absolute value of postoperative SE minus predicted SE). Mean absolute error (MAE), median absolute error (MedAE), and prediction error (PE) were used to compare postoperative refractive outcomes. Auto-refractokeratometer (ARK-510 A, NIDEK, Gamagori, Japan) was used to measure perioperative refractive error. Refractive prediction was considered high if PE was within ± 0.50 D. Negative PE indicates a myopic outcome, and positive PE indicates a hyperopic outcome. The percentages of eyes with PE within ± 0.25 D, ± 0.50 D, ± 0.75 D, and ± 1.00 D were also compared.

### Statistical analysis

SPSS ver.26 (IBM Corp., Armonk, NY, USA) was used for the statistical analysis of data. The normality of the continuous variables was analyzed using the Shapiro–Wilk test. T-test or Mann-Whitney U test was used for subgroup analyses in each formula. The Friedman test was performed to compare the values among the formula within each group, and the Wilcoxon-signed rank test and Bonferroni correction were used for multiple comparisons. For the comparison of proportions, Fisher’s exact test was used. P value less than 0.05 was determined as the level of significance.

## Results

Demographics and preoperative data of study subjects are shown in Table 1. Among 104 eyes of 104 patients, 44 eyes of 44 patients were in the APAC group, and 60 eyes of 60 patients were in the control group. Mean AL, ACD, and ratio of ACD/AL were significantly shorter in the APAC group than the control group (all P < 0.001). The two groups had no significant differences in mean and flat K and WTW (all P > 0.05). Among the APAC group, 31 eyes (70.5%) received an LPI preoperatively. The mean duration when the postoperative refractive power was measured after cataract surgery was 43.5 ± 15.0 days for the APAC and 49.5 ± 17.6 days for the control.

**Table 1:** Demographics and preoperative data of the study subjects

	APAC (n = 44 eyes)			Control (n = 60 eyes)		P value*	
	Mean ± SD	Range		Mean ± SD	Range		
Age (Years)	64.6 ± 9.7	40–82		66.63 ± 6.29	52–82	0.220	
AL (mm)	22.47 ± 0.84	20.26–24.01		23.42 ± 0.80	22.04–25.47	< 0.001	
ACD (mm)	2.45 ± 0.32	1.94–3.23		3.12 ± 0.35	2.12–3.95	< 0.001	
ACD/AL ratio (%)	10.91 ± 1.46	8.45–14.45		13.32 ± 1.41	8.87–15.51	< 0.001	
Flat K (D)	44.16 ± 1.59	40.86–47.67		43.88 ± 1.34	40.71–47.94	0.332	
Steep K (D)	45.31 ± 1.62	42.24–49.34		44.83 ± 1.42	42.35–48.77	0.116	
Mean K (D)	44.74 ± 1.57	41.66–48.47		44.37 ± 1.36	41.77–48.36	0.200	
WTW (mm)	11.57 ± 0.54	10.23–13.10		11.69 ± 0.41	10.63–12.60	0.809	

*T-test was used

D = diopter; SD = standard deviation; APAC = acute primary angle closure; AL = axial length; ACD = anterior chamber depth; K = keratometry; WTW = white to white

**Fig. 1:**
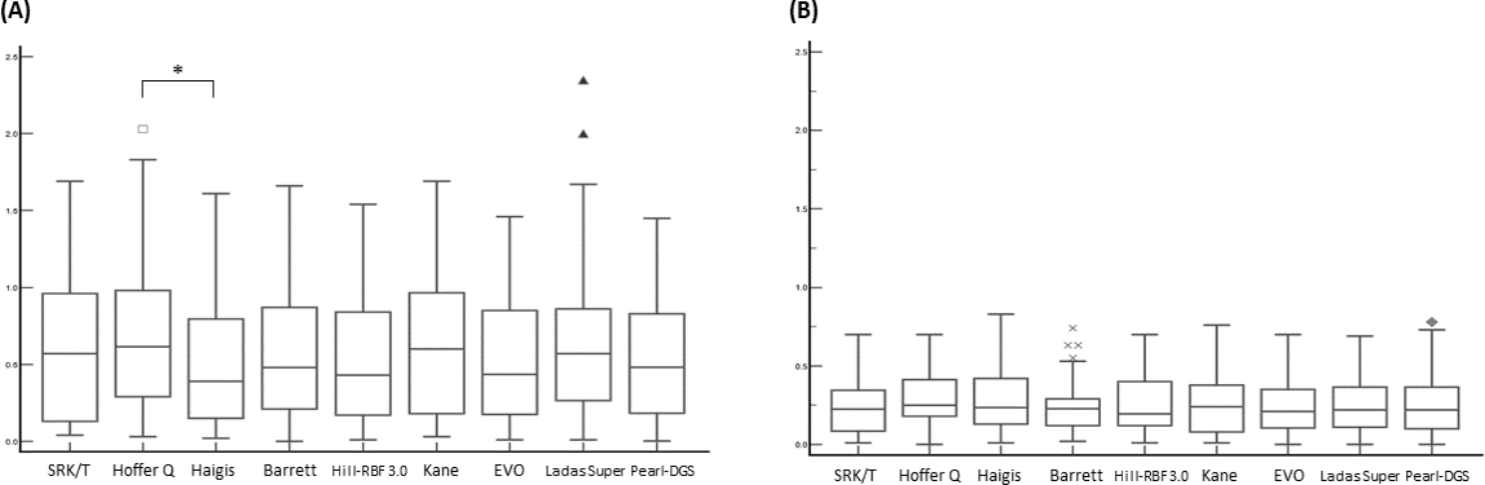
Absolute errors in the APAC (**A**) and the control group (**B**). The central box represents the values from the one to third quartile (25th to 75th percentile). The middle line represents the median value *indicates p value less than 0.05 when using Wilcoxon signed-rank test with Bonferroni post-hoc analysis used to compare the median absolute errors of each IOL formula APAC = acute primary angle closure; Hill-RBF 3.0 = Hill-Radial Basis Function 3.0; EVO = Emmetropia verifying optical; Pearl-DGS = Prediction Enhanced by Artificial Intelligence and output Linearization - Debellemanière, Gatinel, and Saad

Table 2; Fig. 1 show the refractive outcomes in the APAC and control groups. The MedAEs were significantly larger in the APAC group (range, 0.39–0.61 D) compared to the control group (range, 0.19–0.25 D) for all formulas (all P < 0.05). Within the group, there was no statistically significant difference according to the IOL formulas in the control group (P = 0.22). However, in the APAC group, Haigis showed a significantly lower MedAE that Hoffer Q (P = 0.045). The proportion of eyes within ± 0.50 D of PE was lower in the APAC group (45.5–56.8%) than in the control group (85.0-91.7%). In the APAC group, over 10% of eyes (range, 13.6–22.7%) showed a refractive surprise (> 1.0 D), but no eye showed a refractive surprise in the control group.

**Table 2:** Refractive errors obtained with various formulas in the APAC and control groups

	Group	MAE (D)	MedAE (D)	PE ± SD (D)	P value*		Eyes within PE (%)			
							± 0.25 D	±0.50 D	± 0.75 D	± 1.00 D
SRK/T	APAC	0.60	0.57	-0.28 ± 0.73	<0.001		38.6	45.5	63.6	77.3
	Control	0.24	0.22	-0.03 ± 0.31			56.7	86.7	100.0	100.0
Hoffer Q	APAC	0.68	0.61	-0.40 ± 0.75	< 0.001		20.5	45.5	63.6	77.3
	Control	0.29	0.25	0.10 ± 0.33			51.7	88.3	100.0	100.0
Haigis	APAC	0.51	0.39	-0.05 ± 0.67	0.001		36.4	54.6	70.5	86.4
	Control	0.29	0.23	0.09 ± 0.34			53.3	86.7	96.7	100.0
BUII	APAC	0.59	0.48	-0.14 ± 0.77	<0.001		34.1	52.3	70.5	77.3
	Control	0.24	0.23	0.07 ± 0.29			65.0	90.0	100.0	100.0
Hill-RBF 3.0	APAC	0.53	0.43	-0.03 ± 0.69	<0.001		31.8	56.8	70.5	81.8
	Control	0.26	0.19	0.12 ± 0.29			61.7	88.3	100.0	100.0
Kane	APAC	0.64	0.60	-0.33 ± 0.74	< 0.001		29.6	47.7	61.4	79.6
	Control	0.25	0.24	-0.05 ± 0.31			50.0	91.7	98.3	100.0
EVO	APAC	0.55	0.44	-0.09 ± 0.71	< 0.001		36.4	50.0	63.6	84.1
	Control	0.24	0.21	0.06 ± 0.30			61.7	88.3	100.0	100.0
Ladas super	APAC	0.66	0.57	-0.22 ± 0.83	< 0.001		25.0	47.7	68.2	77.3
	Control	0.26	0.22	0.09 ± 0.31			56.7	85.0	100.0	100.0
Pearl-DGS	APAC	0.55	0.48	-0.07 ± 0.71	< 0.001		34.1	52.3	70.5	79.5
	Control	0.26	0.22	0.08 ± 0.31			55.0	90.0	98.3	100.0

*Mann-Whitney U test was used for comparing of absolute errors between the two groups

APAC = acute primary angle closure; MAE = mean absolute error; MedAE = median absolute error; PE = prediction error; SD = standard deviation; Hill-RBF 3.0 = Hill-Radial Basis Function 3.0; EVO = Emmetropia verifying optical; Pearl-DGS = Prediction Enhanced by Artificial Intelligence and output Linearization - Debellemanière, Gatinel, and Saad

Percentages of eyes within ± 0.50 D of PE in each group and the portion of myopia or hyperopia were listed in Table 3. Predictability was higher in the control group than in the APAC group in all IOL formulas (all P < 0.05). In the APAC group, percentages of eyes within ± 0.50 D of PE were between 45.5% (SRK/T and Hoffer Q formula) and 56.8% (Hill-RBF 3.0 formula). In the control group, the percentage of eyes within ± 0.50 D of PE in the control group was 85% or above in all IOL formulas.

**Table 3:** The percentage of the eyes with PE within or outside ± 0.5 D in APAC and control groups

	APAC (%)						Control (%)			P value*
	± 0.50 D	Myopia	Hyperopia			± 0.50 D		Myopia	Hyperopia	
SRK/T	45.5	38.6	15.9			86.7		3.3	10.0	< 0.001
Hoffer Q	45.5	43.2	11.4			88.3		1.7	10.0	< 0.001
Haigis	54.5	25.0	20.5			86.7		1.7	11.7	< 0.001
BUII	52.3	29.5	18.2			90.0		1.7	8.3	< 0.001
Hill-RBF 3.0	56.8	25.0	18.2			88.3		1.7	6.7	< 0.001
Kane	47.7	38.6	13.6			91.7		6.7	1.7	< 0.001
EVO	50.0	29.5	20.5			88.3		3.3	8.3	< 0.001
Ladas super	47.7	31.8	20.5			85.0		1.7	13.3	< 0.001
Pearl-DGS	52.3	27.3	20.5			90.0		1.7	8.3	< 0.001

*Fisher’s exact test

Myopia was defined when PE was more than − 0.50 D and hyperopia was defined when PE was more than + 0.50 D

APAC = acute primary angle closure; Hill-RBF 3.0 = Hill-Radial Basis Function 3.0; EVO = Emmetropia verifying optical; Pearl-DGS = Prediction Enhanced by Artificial Intelligence and output Linearization - dDebellemanière, Gatinel, and Saad

**Fig. 2:**
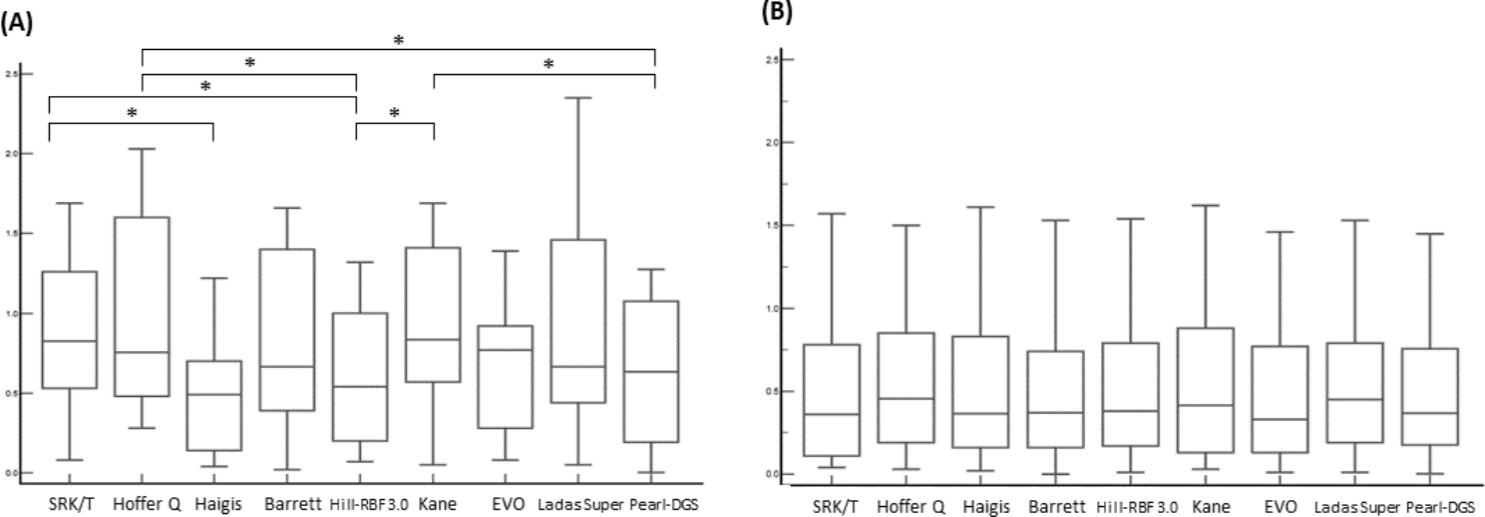
Comparison of median absolute errors in the eyes with AL < 22 mm (**A**) and AL ≥ 22 mm (**B**) in the APAC group. The central box represents the values from the one to third quartile (25th to 75th percentile). The middle line represents the median value *indicates p value less than 0.05 when using Wilcoxon signed-rank test with Bonferroni post-hoc analysis used to compare the median absolute errors of each IOL formula APAC = acute primary angle closure; Hill-RBF 3.0 = Hill-Radial Basis Function 3.0; EVO = Emmetropia verifying optical, Pear DGS = Prediction Enhanced by Artificial Intelligence and output Linearization - Debellemanière, Gatinel, and Saad

To further analyze according to the AL, eyes in the APAC group were divided into two groups: AL < 22 mm (N = 14) and AL ≥ 22 mm (N = 30) (Table 4; Fig. 2). AL and ACD were 21.54 ± 0.45 mm and 2.43 ± 0.29 mm in the AL < 22 mm group and 22.91 ± 0.58 mm and 2.46 ± 0.33 mm in the eyes with AL ≥ 22 mm group. The eyes with AL < 22 mm showed more myopic PE than those with AL ≥ 22 mm. MedAE using SRK/T (P = 0.022) and Hoffer Q (P = 0.023), and Kane (P = 0.010) showed statistical differences between the two AL groups. When comparing the percentage of PE within ± 0.50 D, Haigis and Hill-RBF 3.0 (46.7%) for the AL < 22 mm eyes and BUII and Hill-RBF 3.0 (60.0%) for the AL ≥ 22 mm eyes showed the highest predictability. In the eyes with AL < 22 mm, Haigis showed the lowest MedAE (0.49 D) and SRK/T, Hoffer Q, Kane, and EVO showed 0.75 D or over MedAE. There were statistical inter-formular differences in the AL < 22 mm eyes (P < 0.001). While in the AL ≥ 22 mm eyes, all formulas showed similar MedAE values (range, 0.36–0.45 D, P > 0.05).

**Table 4:** Refractive errors according to the axial length in the APAC group

	AL (mm)	MAE (D)	MedAE (D)	PE ± SD (D)	P value*		Eyes within PE (%)			
							± 0.25 D	± 0.50 D	± 0.75 D	± 1.00 D
SRK/T	< 22	0.85	0.82	-0.75 ± 0.70	0.022		20.0	20.0	46.7	53.3
	≥ 22	0.49	0.36	-0.06 ± 0.65			45.2	54.8	67.7	83.8
Hoffer Q	< 22	0.98	0.75	-0.93 ± 0.68	0.023		0.0	26.7	46.7	53.3
	≥ 22	0.54	0.45	-0.16 ± 0.66			29.0	51.6	67.7	83.9
Haigis	< 22	0.49	0.49	-0.36 ± 0.52	0.846		33.3	46.7	73.3	80.0
	≥ 22	0.52	0.36	0.10 ± 0.68			35.5	54.8	64.5	83.9
BUII	< 22	0.79	0.66	-0.70 ± 0.67	0.121		13.3	33.3	53.3	60.0
	≥ 22	0.50	0.37	0.12 ± 0.68			43.3	60.0	76.7	83.3
Hill-RBF 3.0	< 22	0.60	0.54	-0.45 ± 0.60	0.589		26.7	46.7	60.0	73.3
	≥ 22	0.50	0.38	0.17 ± 0.64			33.3	60.0	73.3	83.3
Kane	< 22	0.91	0.83	-0.80 ± 0.70	0.010		13.3	20.0	46.7	53.3
	≥ 22	0.51	0.41	-0.10 ± 0.66			35.5	58.1	64.5	87.1
EVO	< 22	0.70	0.77	-0.56 ± 0.63	0.131		20.0	33.3	40.0	73.3
	≥ 22	0.48	0.33	0.13 ± 0.64			41.9	54.8	71.0	83.9
Ladas super	< 22	0.94	0.67	-0.85 ± 0.82	0.059		6.7	40.0	53.3	60.0
	≥ 22	0.53	0.45	0.08 ± 0.67			32.3	48.4	71.0	80.7
Pearl-DGS	< 22	0.63	0.63	-0.51 ± 0.59	0.490		26.7	40.0	60.0	66.7
	≥ 22	0.52	0.37	0.13 ± 0.68			36.7	56.7	73.3	83.3

AL < 22 mm (N = 14) and AL ≥ 22 mm (N = 30)

*Mann-Whitney U test was used for comparing of absolute errors between the two groups

MAE = mean absolute error; MedAE = median absolute error; PE = prediction error; SD = standard deviation; Hill-RBF 3.0 = Hill-Radial Basis Function 3.0; EVO = Emmetropia verifying optical; Pearl-DGS = Prediction Enhanced by Artificial Intelligence and output Linearization - Debellemanière, Gatinel, and Saad

To identify whether preoperative LPI affects refractive outcomes in the APAC group, MedAEs were compared between the non-iridotomized eyes (N = 13) and the iridotomized eyes (N = 31) (Supp Table 1). Thirteen eyes did not perform LPI because of severe corneal edema, shallow anterior chamber, or inadequate miosis. AL was 22.21 ± 0.78 mm in the non-iridotomized eyes and 22.58 ± 0.85 mm in the iridotomized eyes, and there was no difference between the two groups (P = 0.190). The mean ACD was 2.39 ± 0.24 mm in the non-iridotomized eyes and 2.47 ± 0.35 mm in the iridotomized eyes, and there was no difference between the two groups (P = 0.430). MedAEs in the iridotomized eyes showed a lower tendency (range, 0.36–0.61 D) than those in the non-iridotomized eyes (range, 0.48–0.73 D), but there were no statistical differences (all P > 0.05).

## Discussion

This study compared the refractive outcomes of nine IOL formulas in patients with APAC history. In APAC eyes, inferior predictability and myopic shift were observed compared to the control eyes. MedAEs were not statistically different in APAC eyes of AL ≥ 22 mm; however, in APAC eyes with AL < 22 mm, Haigis and Hill-RBF 3.0 showed the lowest MedAEs. Preoperative LPI did not affect the postoperative refractive outcomes in APAC eyes.

Previous studies using conventional IOL formulas reported that eyes with a history of PACG yield inaccuracy in refractive prediction [1, 7, 12–14]. Joo et al. [12] compared the refractive outcome after the cataract surgery in 63 eyes with APAC who received preoperative LPI and 93 eyes in the control group for three traditional formulas (Hoffer Q, SRK/T, and Haigis). The authors reported that Haigis showed inferior predictability (MAE, 0.69 ± 0.54 D) and Hoffer Q (MAE, 0.53 ± 0.39 D) showed superior outcomes than the others in PACG patients. A more hyperopic shift was observed in the SRK/T and Haigis formulas and a myopic shift in the Hoffer Q formula. Seo et al. [6] reported that among 193 PAC or PACG patients, Hoffer Q (0.44 ± 0.34 D) had the least MPE among Hoffer Q, Haigis (0.50 ± 0.37 D), and SRK/T (0.46 ± 0.34 D) formulas. A hyperopic shift was observed in all formulas. In contrast, Day et al. [8] reported that Haigis showed the lowest MAE (0.30 ± 0.20 D) and Hoffer Q showed the highest MAE (1.11 ± 1.32 D) among Hoffer Q, SRK/T, and Haigis formulas in the effectiveness in angle-closure glaucoma of lens extraction study (EAGLE) (N = 208), in line with this study’s results. Day et al.’s study observed a myopic shift for Hoffer Q and a hyperopic shift for SRK/T. Meanwhile, Lee et al. [1] reported that peripheral anterior synechia (PAS) is related to higher MAE and myopic shift regardless of IOL formulas, including SRK/T, Hoffer Q, Haigis, and Holladay.

There are several hypotheses regarding postoperative refractive outcomes in PAC or PACG patients. The myopic shift may be associated with a high anterior lens vault, defined as the perpendicular distance between the anterior pole of the lens and a horizontal line between the scleral spurs, resulting in an anteriorly positioned IOL after surgery [14, 15]. In contrast, hyperopic shift may also occur because cataract surgery yields deepening of the anterior chamber and IOL shift to a more posterior plane by removing the lens volume and the pupillary block component. Decreased AL because of IOP lowering also can contribute to hyperopic shift [6]. If PAS is present, PAS may interfere with ciliary body reposition and posterior shifting of the IOL plane, also resulting in myopic shift [1]. In this study, APAC eyes showed more myopic shift than control groups. We did not evaluate the preoperative lens vault using anterior segment optical coherence tomography, and the status of PAS was not accessed in all eyes, so the exact mechanism of myopic shift was not suggested in this study.

Efforts to improve the postoperative outcome of cataract surgery are in place with the development of various formulas, including artificial intelligence. New generation formulas such as BUII, Kane, or Hill-RBF 3.0 showed higher postoperative accuracy than the conventional formulas in cataractous eyes [16–21]. Until now, two studies by Hou et al. [7] and Ding et al. [11] analyzed accuracy using recent IOL formulas for primary angle-closure disease (PACD). Hou et al. evaluated the accuracy of seven IOL formulas (SRK/T, Haigis, Hoffer Q, BUII, Kane, Ladas super formula, and Hill-RBF 3.0) for the five different IOL models including ZCB00 (54%), MX60 (27.9%), SN60WF (13.2%), Aspira-aA (9.3%), and 970 C (7.8%). About two-thirds of the 129 eyes of 129 patients (N = 80) received additional glaucoma surgeries such as peripheral iridectomy and trabeculectomy in addition to cataract surgery. In their study, Kane and Hill-RBF 3.0 formulas performed better in new-generation formulas, while Haigis and SRK/T formulas showed better results in traditional formulas. The Kane formula showed the best accuracy in PACD with shorter AL (< 22 mm). On the contrary, Haigis and Hill-RBF 3.0 formulas showed better predictability in the APAC group in this study, while Hoffer Q, Kane, and Ladas super showed worse accuracy. In a study recently conducted by the same research group [11] compared six IOL formulas (BUII, Haigis, Hill-RBF 3.0, Hoffer Q, Kane, and SRK/T) in patients with PACD using single type of IOL and concluded that the standard deviation of Kane (0.59), Hill-RBF 3.0 (0.61) and SRK/T (0.62) were significantly lower than Hoffer Q (0.68). Study results of the two studies are somewhat different from ours: the formula showing the lowest standard deviation in the APAC group for our study was Haigis (0.67), followed by Hill-RBF 3.0 (0.69) which were slightly larger than those in the Ding et al.’s study and the percentage of eyes within PE ± 0.5 D was much higher (60.5 to 71.3%) in Hou’s study than those in APAC eyes in this study (45.5 to 56.8%). The difference may be related to the difference in ACD among the studies, which is shorter in Hou et al.’s study (2.22 ± 0.26 mm for no-surgery patients, 2.25 ± 0.35 mm for peripheral iridectomy patients, and 2.3 ± 0.32 mm for trabeculectomy patients) and Ding et al.’s study (2.21 ± 0.28 mm) than those in this study (2.43 ± 0.29 mm for eyes with AL < 22 mm and 2.46 ± 0.33 mm for eye with AL ≥ 22 mm).

AL and ACD are known to be important factors that affect the prediction of refractory outcomes. There have been many reports about IOL power calculation in short eyes [14, 15, 22–27]. In a study by Carmona-Gonzalez et al. [28], Haigis and EVO seem to be accurate when compared to the other ten formulas, including SRK/T, Hoffer Q, Holladay I, Holladay II, Haigis, Olsen, BUII, Hill-RBF, Ladas Super formula, and Kane. Eom et al. [23] reported that Haigis is more accurate than Hoffer Q when ACD was less than 2.40 mm. Shirvastava et al. [24] concluded that among seven formulas, Haigis showed the best outcome in short eyes with ACD greater than 2.40 mm (BUII, Haigis, Hill-RBF, Hoffer Q, Holladay I, Holladay 2, and SRK/T). This study also showed similar results to these studies. In the APAC group that showed short AL < 22 mm with shallow ACD (2.43 **±** 0.29 mm), the Haigis showed the lowest MedAE values.

LPI relieves the pressure acting forward on the lens, resulting in the deepening of the central anterior chamber and backward positioning of the lens [29, 30]. Therefore, LPI may stabilize the effective lens position by deepening the ACD with the shortening of AL [13]. However, previous studies reported that ACD did not change after LPI [31, 32], and the effect of LPI on angle widening in APAC depends on PAS [30, 33]. This study also noted no statistical significance between those two groups, indicating that preoperative LPI does not benefit postoperative refractive outcomes. Further analysis with a larger cohort would be necessary to conclude the effect of LPI on postoperative refractive outcomes in APAC patients.

This study has some limitations. First, we did not clarify the angling status in all APAC patients. Of the 44 eyes in the APAC group, nine had no data of gonioscopy on preoperative status because they were transferred to our hospital from another clinic for cataract surgery after initial management, including LPI. The existence of PAS was difficult to evaluate in two eyes due to failure to relieve appositional obstruction of the trabecular meshwork during gonioscopy with indentation. Only one eye had PAS in the APAC group. The existence of PAS may induce higher MAE and more myopic shift, a lower proportion of PAS in this study would be considered when interpreting the results [6]. Second, only Asian patients were included in this study. Because there are ethnic differences in corneal curvature, anterior chamber angle, and AL [34], further study would be necessary to expand this study’s results in patients of other ethnicities. Third, while measuring the refraction by subjective refraction is the gold standard, the refractive error of the patients was measured with an auto-refractokeratometer. Fourth, LT or central corneal thickness may contribute to the accuracy of the formula [35, 36], these parameters were not considered in this study. Fifth, we could not fully optimize the lens constants for every formula since the status of APAC eyes, including ACD and ACD/AL ratio, is quite different from each other, and the study results were summed of three experienced but different surgeons. Further study using optimized lens constant for APAC patients calculated from a large cohort would be necessary.

In conclusion, compared to the control eyes APAC eyes showed myopic refractive outcomes. Haigis and Hill-RBF 3.0 showed high precision in the eyes with AL < 22 mm in the APAC group. None of the formulas shows statistically superior refractive outcomes in eyes with AL ≥ 22.0 mm in the APAC patients.

### Electronic supplementary material

Below is the link to the electronic supplementary material.

Supplementary Material 1
