# Supplementary material for: Comparison of intraocular lens power calculation formulas in patients with a history of acute primary angle-closure attack
Source: BMC Ophthalmol. 2023 Nov 24;23:482. doi: 10.1186/s12886-023-03232-5 (PMC10675974; doi:10.1186/s12886-023-03232-5)
Supplement: Supplementary file 1 — Supplementary Material 1 [file 12886_2023_3232_MOESM1_ESM.docx]

**Supp Table 1. Refractive errors between iridotomized and non-iridotomized eyes in the APAC group.**

|  | **LPI** | **MAE (D)** | **MedAE (D)** | **PE ± SD (D)** | **P value*** |  | **Eyes within PE (%)** | | | |
| --- | --- | --- | --- | --- | --- | --- | --- | --- | --- | --- |
|  |  |  |  |  |  |  | **±0.25 D** | **±0.50 D** | **±0.75 D** | **±1.00 D** |
| SRK/T | + | 0.58 | 0.54 | -0.27 ± 0.71 | 0.554 |  | 38.7 | 48.4 | 67.7 | 80.7 |
|  | - | 0.67 | 0.65 | -0.32 ± 0.83 |  |  | 38.5 | 38.5 | 53.9 | 61.5 |
| Hoffer Q | + | 0.66 | 0.61 | -0.38 ± 0.75 | 0.291 |  | 19.4 | 45.2 | 71.0 | 83.9 |
|  | - | 0.75 | 0.73 | -0.47 ± 0.80 |  |  | 23.1 | 46.2 | 46.2 | 53.9 |
| Haigis | + | 0.48 | 0.36 | -0.02 ± 0.63 | 0.965 |  | 41.9 | 58.1 | 67.7 | 87.1 |
|  | - | 0.59 | 0.51 | -0.11 ± 0.79 |  |  | 23.1 | 46.2 | 76.9 | 76.9 |
| BUII | + | 0.56 | 0.48 | -0.13 ± 0.75 | 0.620 |  | 34.4 | 53.1 | 78.1 | 81.3 |
|  | - | 0.67 | 0.61 | -0.18 ± 0.86 |  |  | 33.3 | 50.0 | 50.0 | 66.7 |
| Hill-RBF 3.0 | + | 0.50 | 0.43 | 0.00 ± 0.65 | 0.577 |  | 37.5 | 59.4 | 71.9 | 84.4 |
|  | - | 0.62 | 0.48 | -0.09 ± 0.81 |  |  | 16.7 | 50.0 | 66.7 | 75.0 |
| Kane | + | 0.61 | 0.60 | -0.29 ± 0.72 | 0.448 |  | 29.0 | 48.4 | 67.7 | 87.1 |
|  | - | 0.72 | 0.66 | -0.42 ± 0.83 |  |  | 30.8 | 46.2 | 50.0 | 53.9 |
| EVO | + | 0.53 | 0.44 | -0.08 ± 0.69 | 0.964 |  | 38.7 | 48.4 | 67.7 | 83.9 |
|  | - | 0.62 | 0.56 | -0.10 ± 0.79 |  |  | 23.1 | 46.2 | 50.0 | 76.9 |
| Ladas super | + | 0.63 | 0.51 | -0.19 ± 0.83 | 0.473 |  | 25.8 | 51.6 | 74.2 | 80.7 |
|  | - | 0.73 | 0.73 | -0.27 ± 0.88 |  |  | 23.1 | 38.5 | 50.0 | 61.5 |
| Pearl-DGS | + | 0.53 | 0.48 | -0.06 ± 0.68 | 0.463 |  | 40.6 | 53.1 | 71.9 | 81.3 |
|  | - | 0.62 | 0.50 | -0.11 ± 0.81 |  |  | 16.7 | 50.0 | 66.7 | 75.0 |

Iridotomized eyes (N=31) and virgin eyes (N=13)

*Mann-Whitney U test was used for comparing of absolute errors between the two groups.

APAC = acute primary angle closure; LPI = laser peripheral iridotomy; MAE = mean absolute error; MedAE = median absolute error; PE = prediction error; SD = standard deviation; Hill-RBF 3.0 = Hill-Radial Basis Function 3.0; EVO = Emmetropia verifying optical; Pearl-DGS = Prediction Enhanced by Artificial Intelligence and output Linearization - Debellemanière, Gatinel, and Saad
